# Inflammation and Infection in Pain and the Role of GPR37

**DOI:** 10.3390/ijms232214426

**Published:** 2022-11-20

**Authors:** Qin Zhang, Sangsu Bang, Sharat Chandra, Ru-Rong Ji

**Affiliations:** 1 Department of Anesthesiology, Duke University Medical Center, Durham, NC 27710, USA; 2 Center for Translational Pain Medicine, Department of Anesthesiology, Duke University Medical Center, Durham, NC 27710, USA; 3 Department of Neurobiology, Duke University Medical Center, Durham, NC 27710, USA; 4 Department of Cell Biology, Duke University Medical Center, Durham, NC 27710, USA

**Keywords:** docosahexaenoic acid (DHA), GPR37, infection, inflammation, macrophages, neuroprotectin D1 (NPD1), malaria, pain, sepsis, specialized pro-resolving mediator (SPM)

## Abstract

Inflammation is known to cause pain, and pain is of one of the cardinal signs of inflammation. Mounting evidence suggests that acute inflammation also resolves pain through specialized pro-resolving mediators (SPMs) and macrophage signaling. GPR37 is expressed by neurons and oligodendrocytes in the brain and has been implicated in multiple disorders, such as demyelination, Parkinson’s disease, stroke, and cancer. Recent studies have demonstrated that GPR37 is expressed by macrophages and confers protection against infection by bacteria and parasites. Furthermore, GPR37 promotes the resolution of inflammatory pain and infection-induced pain, as the duration of pain after tissue injury and infection is prolonged in mice lacking *Gpr37*. Mechanistically, activation of GPR37 enhances macrophage phagocytosis, and *Gpr37*-deficient macrophages exhibit dysregulations of pro-inflammatory and anti-inflammatory cytokines, switching from M2- to M1-like phenotypes. We also discuss novel ligands of GPR37, including neuroprotectin D1 (NPD1), a SPM derived from docosahexaenoic acid (DHA), and bone-derived hormone osteocalcin (OCN), which can suppress oligodendrocyte differentiation and myelination. NPD1 stimulates macrophage phagocytosis via GPR37 and exhibits potent analgesic actions in various animal models of inflammatory and neuropathic pain. Targeting GPR37 may lead to novel therapeutics for treating inflammation, infection, pain, and neurological diseases.

## 1. Introduction

Inflammation is characterized by five cardinal signs: *rubor* (redness), *calor* (increased heat), *tumor* (swelling), *dolor* (pain), and *functio laesa* (loss of function). Pain after inflammation aims to protect the inflamed tissue by sending the nociceptive warning signal to the brain, triggering withdrawal and emotional/affective responses. Pain is sensed by specialized primary sensory neurons in the peripheral nervous system (PNS), named nociceptors [1]. The 2021 Nobel Prize in Physiology or Medicine was awarded for the discovery of molecular sensors of pain, including the heat pain sensor TRPV1 (transient receptor potential ion channel subtype V1) and the mechanical pain sensor Piezo2 [2]. Activation of nociceptors causes neurogenic inflammation by releasing neuropeptides such as substance P and calcitonin gene-related peptide (CGRP) [3,4], as well as chemokines (e.g., CCL2) [5]. However, ablation of nociceptive neurons may potentiate or inhibit inflammation in a context-dependent manner [6,7]. Inflammation after tissue injury results in sequential infiltration of immune cells into the damaged tissue, including neutrophils within hours and macrophages within a day, as well as T cells within days to weeks [8,9]. The infiltrating and activated immune cells produce inflammatory mediators such as proinflammatory cytokines (TNF, IL-1β, IL-17) and chemokines; these mediators have been shown to induce pain by binding to cytokine/chemokine receptors on nociceptors throughout skin, muscle, and joint tissues [10,11,12]. Some inflammatory mediators such as CXCL5 and IL-23 may indirectly promote pain via their actions on immune cells, which subsequently activate nociceptors via releasing additional inflammatory mediators (e.g., IL-17 from macrophages) [12,13,14]. 

The initial purpose of inflammation is to remove the cause of infections and cell injury and promotes tissue repair and wound healing. Resolution of acute inflammation, once regarded as a passive process, is now being appreciated as an active program that depends on the production of specialized pro-resolving mediators (SPMs) for the control of inflammation and pain. Importantly, blockade or disruption of this pro-resolution program may lead to chronic inflammation and chronic pain in many disease conditions [15,16,17]. SPMs, such as resolvins, protectins, and maresins, are biosynthesized from omega-3 unsaturated fatty acids (e.g., docosahexaenoic acid, DHA). A large body of preclinical studies suggests that administration of synthetic SPMs can alleviate inflammatory pain at very low doses (1–100 ng), which are much lower than that of morphine, a potent opioid analgesic that also produces detrimental side effects, such as addiction, respiratory suppression, and constipation [9,18,19,20]. DHA-derived protectin D1, also called neuroprotectin D1 (NPD1) due to its brain-protective actions [21,22], induces potent inhibition of inflammatory and neuropathic pain [23,24]. SPMs are known to activate G-protein coupled receptors (GPCR) to meditate their pro-resolution actions [18,25,26] through multiple cell types, including immune and glial cells, as well as neurons [20]. In addition to treating inflammatory pain [27], synthetic SPMs have also been shown to alleviate neuropathic pain, cancer pain, and postoperative pain [20,28,29]. 

A key mechanism for SPMs to resolve inflammation is to promote phagocytosis, leading to removal of pathogens and cell debris after inflammation [30,31]. Notably, macrophages play a critical role in phagocytosis [32,33,34] and display different phenotypes, including M1-like pro-inflammatory and M2-like anti-inflammatory phenotypes [33]. Using multiple in vitro and in vivo approaches, as well as computer simulations, we recently identified GPR37 as a novel receptor for NPD1 that regulates macrophage phagocytosis and changes macrophage phenotypes [35].

Recent progress has shown an important role of GPR37 in immune cells. Activation of GPR37 promotes macrophage phagocytosis and resolution of inflammation [35,36]. In this review, we discuss the beneficial and detrimental roles of GPR37 in neurons, glial cells, cancer cells, and immune cells. In particular, we will highlight novel ligands such as NPD1 [35,36] and osteocalcin [37] and highlight new drug discovery opportunities for targeting GPR37.

## 2. GPR37 in Health and Disease

**Figure 1:**
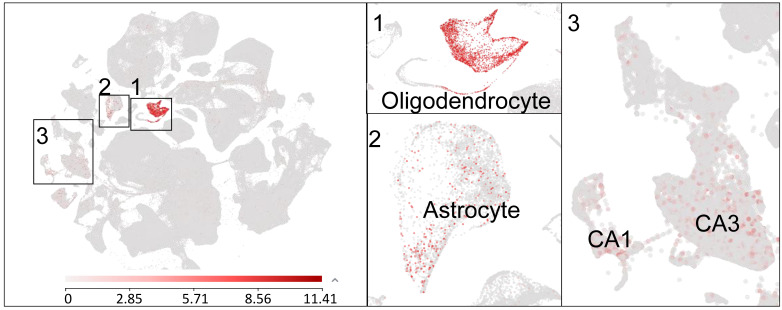
Single-cell RNAseq showing GPR37 expression in the mouse brain. A meta-analysis reveals GPR37 mRNA expression in oligodendrocytes (box 1), astrocytes (box 2), and hippocampal CA1 and CA3 neurons (box 3). The small boxes are enlarged in the middle and right panels. The data are plotted from the Allen brain map dataset (Mouse-Whole cortex & Hippocampus-10X). (https://celltypes.brain-map.org/rnaseq/mouse_ctx-hpf_10x?selectedVisualization=Scatter+Plot&colorByFeature=Gene+Expression&colorByFeatureValue=Gpr37, accessed on 21 October 2021).

GPR37, also known as parkin-associated endothelin-like receptor (Pael-R), was cloned in 1997 and was initially implicated in Parkinson’s disease (PD) as well as in autism [38,39]. GPR37 is a substrate of parkin, and, notably, insoluble aggregates of GPR37 are accumulated in Lewy bodies in brain samples of PD patients [40]. Mutations in *GPR37* are associated with autism spectrum disorders [41]. GPR37 is highly expressed by oligodendrocytes in the brain and spinal cord and regulates the function of these glial cells in the central nervous system (CNS) [42]. GPR37 is a negative regulator of oligodendrocyte differentiation and myelination, and, strikingly, mice lacking *Gpr37* display hypermyelination [37,42]. Prosaposin and prosaposin-derived 14-mer peptide (TX14) were initially identified as ligands of GPR37 and GPR37L1, a close family member of GPR37, and exhibit neuroprotective and analgesic effects [43,44]. However, the binding sites of prosaposin and TX-14 for GPR37 and GPR37L1 were not demonstrated, and these peptides have not been vigorously tested in *Gpr37* and *Gpr37l1* knockout mice. Single cell RNAseq reveals *Gpr37* mRNA expression in several cell types in the mouse brain, including oligodendrocytes and astrocytes, as well as the CA1/CA3 region of hippocampal neurons. In particular, *Gpr37* mRNA is highly expressed in oligodendrocytes in the CNS (Figure 1). 

As expected, GPR37 plays an important role in oligodendrocyte differentiation and myelination. GPR37 expression increases in oligodendrocytes during their differentiation into myelin-forming cells. Genetic deletion of *Gpr37* does not affect the number of oligodendrocyte precursor cells (OPC) but impairs the differentiation of oligodendrocytes [42]. Mice lacking *Gpr37* exhibit decreased expression of the myelin-associated glycoprotein MAG and increased susceptibility to demyelination. In the cuprizone model of demyelination, *Gpr37*^−/−^ mice display dramatic loss of myelin in response to cuprizone [45]. Thus, GPR37 appears to be protective against the demyelination that occurs in various neurological diseases (Table 1). Notably, the bone-derived hormone osteocalcin, which plays a critical role in brain development and cognition, was identified as a novel ligand of GPR37. Loss of osteocalcin was shown to facilitate oligodendrocyte differentiation and cause hypermyelination in the CNS [37], which mimics the phenotypes of *Gpr37*^−/−^ mice. Osteocalcin is also required for remyelination after lysolecithin-induced demyelination [37].

GPR37 has been implicated in several neurological disorders, such as PD and stroke, and in cancer (Table 1). Parkin is an E3 ubiquitin ligase that specifically recognizes its substrate proteins, promoting their ubiquitination and subsequent degradation. Mutations in parkin results in loss of its ubiquitination E3 ligase activity and failure to appropriately remove the accumulated substrates, including GPR37. In 2001, the G-protein-coupled transmembrane polypeptide Pael-R was identified as an interacting protein with parkin [46]. Pael-R is accumulated in the Lewy bodies of PD patients [40] and triggers the unfolded protein response, leading to dopaminergic neuronal cell death. GPR37 and GPR37L1 may form homodimers or heterodimers in live N2a cells. GPR37 was identified in the cytoplasm, and this expression could be counteracted by overexpression of parkin [47]. Importantly, GPR37 can be both detrimental and protective, depending on its cellular location. Disruption of GPR37 and parkin interaction by small interfering RNA was shown to induce GPR37 intracellular accumulation and caspase-3 activation, leading to apoptosis [48]. In cell viability study with neurotoxin challenge, GPR37 is functionally trafficked to the plasma membrane to protect against cell toxicity [49]. GPR37 expression is also found to increase in the substantia nigra of sporadic PD patients. Furthermore, the ecto-GPR37 peptides are significantly increased in the cerebrospinal fluid (CSF) of PD patients but not increased in the CSF of Alzheimer’s disease patients, suggesting that ecto-GPR37 may serve as a potential biomarker for PD [50]. Mutations in GPR37 were related to the deleterious effect of autism spectrum disorder, a neurodevelopmental and neuropsychiatric disorder [41]. Therefore, GPR37 may be involved in multiple neurological diseases. 

Recent study revealed a protective role of GPR37 in stroke (Table 1). In an ischemic stroke model, *Gpr37*^−/−^ mice exhibited increased infarction and cell death compared with wild-type mice, which is associated with significantly more apoptotic and autophagic cell death [51]. Following stroke injury, GPR37 is increased within a population of Sox2-positive progenitor cells and acts as a negative regulator of progenitor cell dynamics and gliosis following ischemic injury [52]. 

Several studies have shown a detrimental role of GPR37 in promoting tumor growth (Table 1). In human lung adenocarcinoma, GPR37 is upregulated and associated with a poor prognosis. GPR37 downregulation markedly inhibited the proliferation and migration of the tumor cells both in vitro and in vivo by binding to protein kinase CDK6. GPR37 further facilitates tumorigenesis of xenograft tumors in vivo [53]. GPR37 is identified to be in the same complex of REG4 (regenerating islet-derived family, member 4) and mediates REG4’s signal transduction, promoting peritoneal metastasis of gastric cancer cell [54]. GPR37 expression is low in multiple myeloma cell adhesion models and high in proliferating cells. In vitro meddling with the expression of GPR37 altered the activity of Akt and ERK in multiple myeloma cells [55]. Immunohistochemistry and Western blot analyses in hepatocellular carcinoma samples revealed low expression of GPR37 in hepatocellular carcinoma (HCC) as compared to non-tumorous tissues nearby. Pathological analysis showed that GPR37 expression was correlated with histological grade and alpha fetal protein level. However, it was also revealed that decreasing GPR37 expression was associated with poor prognosis in HCC patients. Thus, GPR37 can either be detrimental or protective in liver cancer [56].

**Table 1:** Beneficial and detrimental roles of GPR37 in different disease conditions.

Disorders	Cell Types	Major Findings	Protective Detrimental	References
Parkinson’s Disease	Neuron	GPR37 (Pael-R) accumulation in Lewy bodies associated with increased death of dopaminergic neurons	Detrimental	[40]
		Disruption of GPR37 and parkin interaction induces GPR37 intracellular accumulation and apoptosis		[47]
Autism Spectrum Disorder (ASD)	Neuron	*GPR37* mutations on chromosome 7q31-33 are associated with ASD	Protective	[41]
Brain Stroke	Neuron	*Gpr37* knockout mice have increased infarctions and cell death	Protective	[51]
		GPR37 negatively regulates gliosis following ischemic injury		[52]
Demyelination	Oligodendrocytes	*Gpr37* knockout mice show increased susceptibility to demyelination	Protective	[42]
Human Lung Adenocarcinoma	Tumor Cells	Upregulation of GPR37 is correlated with poor prognosis	Detrimental	[53]
Multiple Myeloma	Tumor Cells	High expression of GPR37 in proliferating cells	Detrimental	[55]
Hepatocellular Carcinoma	Tumor Cells	Low expression in tumor tissue and decreasing GPR37 expression is associated with poor prognosis in HCC patients	Protective	[56]
Bacterial Infections/ Sepsis	Macrophage	NPD1 and ARU increase macrophage phagocytosis via GPR37	Protective	[14]
		NPD1 and GPR37 protects infection by listeria and sepsis by LPS		[36]
		Osteocalcin protects LPS-induced sepsis		[36]
Malaria	Macrophage	NPD1 and ARU increase macrophage phagocytosis of infected red blood cells	Protective	[36]
Inflammatory Pain	Macrophage	Inflammatory pain is prolonged in *Gpr37* knockout mice	Protective	[35,36]
Infection-Induced Pain		Infection-induced pain is prolonged in *Gpr37* knockout mice, ARU inhibits infection pain		

## 3. GPR37 Signaling in Macrophages in Inflammation and Inflammatory Pain

**Figure 2:**
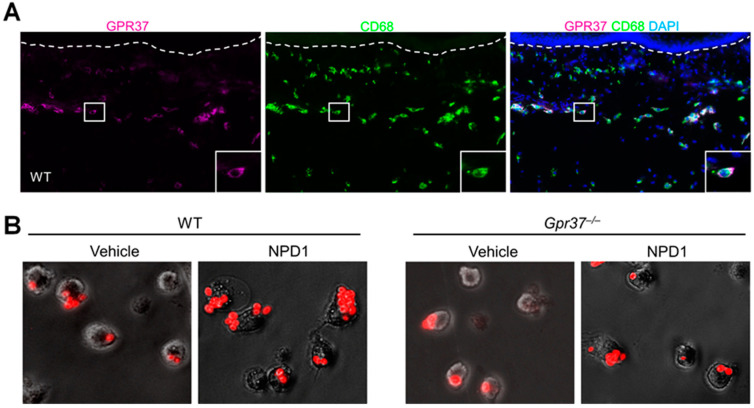
GPR37 expression in macrophages promotes macrophage phagocytosis. (**A**) Immunohistochemistry reveals the co-expression of GPR37 with activated macrophage marker CD68 in the dermis of hindpaw skin of WT mice. (**B**) Macrophage phagocytosis of zymosan particles. NPD1 enhanced phagocytosis of zymosan particles in WT peritoneal macrophages. NPD1-induced macrophage phagocytosis is diminished in *Gpr37*^−/−^ mice. There figures are reproduced from Bang et al., JCI, 2018 with permission [35].

GPR37 expression was examined in normal and inflamed skin using immunohistochemistry, flow cytometry, and in situ hybridization. GPR37 protein is present in the dermis of hindpaw skins of wild-type mice, but this expression was not found in the skin of knockout mice lacking *Gpr37* (*Gpr37*^−/−^ mice). Furthermore, *Gpr37* mRNA is co-expressed with GPR37 protein in the dermis [35]. GPR37 expression in macrophages was validated by co-localization of GPR37 with CD68, a cellular marker for activated macrophages (Figure 2A). Flow cytometry demonstrated that GPR37 was expressed in 30–65% of macrophages. Surface and cytoplasm localization of GPR37 in peritoneal macrophages was demonstrated by confocal microscopy. GPR37 is also expressed by F4/80^+^ macrophages in DRG where sensory neurons are localized. 

GPR37 signaling in macrophages has been revealed by Ca^2+^ imaging and phagocytotic assay. In heterologous HEK293 cells over-expressing GPR37, activation of this receptor by TX14 (1 μM) and NPD1 (30 nM) evoked marked increases in intracellular Ca^2+^. Notably, other SPMs such as resolvins and their precursors (DHA and EPA) failed to induce intracellular Ca^2+^ signaling. NPD1 also triggered Ca^2+^ increase in native macrophages. Mechanistically, the intracellular increase in Ca^2+^ triggers macrophage phagocytosis of zymosan, a pathogen that activates TLR2 to induce inflammation and pain [57]. Using pH-sensitive zymosan particles, which show fluorescence only after phagocytosis, we observed that NPD1 treatment significantly increased the phagocytic activity of cultured peritoneal macrophages. This effect is GPR37-mediated, as it was only observed in wild-type macrophages but not in *Gpr37*-deficient macrophages (Figure 2B). 

**Figure 3:**
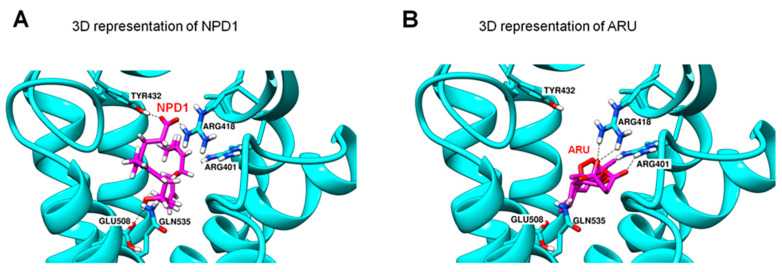
3D representation of NPD1 (**A**) and ARU (**B**) interactions with GPR37. The docking results show postulated GPR37 binding sites for NPD1 and the antimalaria drug artesunate (ARU). NPD1 may form hydrogen bonds with four GPR37 residues: R418, Y432, E508, and Q535 residues. ARU may form a hydrogen bond with R401, R418, and Q535. It is noteworthy that the residues R418 and Q535 may interact with both NPD1 and ARU. This figure is reproduced from Bang et al., Nat Commun, 2021 with permission [36]. This is an open access article under the Creative Commons CC BY license.

GPR37 is also involved in macrophage phagocytosis of apoptotic neutrophils in inflamed skin. Lipid overlay and dot blot assays revealed NPD1 and TX-14 binding to GPR37, but the binding affinity of NPD1 is higher than TX-14 [35]. Computational modeling revealed possible interactions between NPD1 and GPR37 (Figure 3). Molecular dynamics simulations suggest that NPD1 can form hydrogen bonds with several amino acid residues on GPR37, including E508, Q535, R418, and Y432 (Figure 3A). However, TX-14 does not share the same NPD1 binding sites on GPR37. Instead, the second extracellular loop (ECL2) of GPR37 could be a putative binding site for TX-14. It is likely that NPD1 and TX-14 interact with GPR37 via distinct binding sites and, therefore, differentially regulate the activity of GPR37. 

GPR37 also regulates macrophage phenotypes. Loss of GPR37 in peritoneal macrophages leads to phenotype changes, switching from M2-like macrophages to M1-like macrophages. *Gpr37*-deficient macrophages produced increased levels of pro-inflammatory cytokines (e.g., IL-1β, TNF, IL-6) but decreased levels of anti-inflammatory cytokines (IL-10 and TGF-β1) in response to inflammatory challenge. In contrast, NPD1 treatment increased the levels of IL-10 and TGF-β1 but decreased the levels of IL-1β, TNF, and IL-6 [36]. 

**Figure 4:**
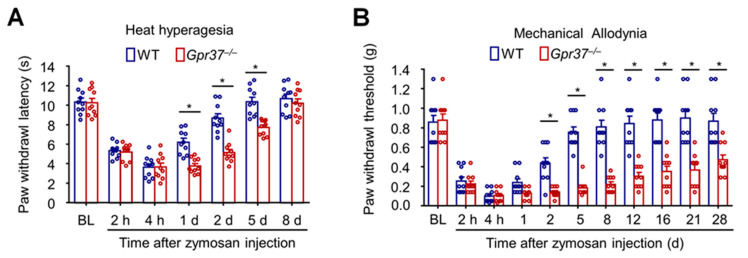
Zymosan-induced inflammatory pain in WT and *Gpr37* KO mice. (**A**) Heat hyperalgesia. (**B**) Mechanical allodynia. *Gpr37*^−/−^ mice exhibit intact baseline pain, and the onset of inflammatory pain were also normal in these mice. However, the resolution/recovery phase of inflammatory pain was prolonged in KO mice. * *p* < 0.05, compared to *Gpr37*^−/−^. The data were analyzed with two-way ANOVA. n = 10 mice per group. BL, baseline. These graphs are reproduced from Bang et al., JCI, 2018 with permission [35].

GPR37 plays a crucial role in resolving inflammatory pain [35]. Inflammatory pain is typically measured by heat hyperalgesia, revealed by reduction in paw withdrawal latency in response to radiant heat stimulation, and mechanical allodynia, revealed by reduction in paw withdrawal threshold in response to von Frey hair stimulation. Zymosan not only activates immune cells (e.g., macrophages) but also results in edema and inflammatory pain. Intraplantar injection of zymosan in a mouse hindpaw elicited both heat hyperalgesia and mechanical allodynia in WT mice. Compared with WT mice, the baseline pain and the induction phase of inflammatory pain (assessed by heat hyperalgesia and mechanical allodynia) were not impacted in *Gpr37*^−/−^ mice. However, the recovery of inflammatory pain is impaired in *Gpr37*^−/−^ mice. The duration of heat hyperalgesia is increased from 2 days in WT mice to more than 1 week in *Gpr37*^−/−^ mice, and the duration of mechanical pain is extended from 1 week in WT mice to 1 month in *Gpr37*^−/−^ mice (Figure 4A,B). 

As a primary pro-inflammatory cytokine, IL-1β plays an active role in driving inflammatory pain [11]. Hindpaw injection of IL-1β is sufficient to evoke both heat hyperalgesia and mechanical allodynia in WT mice, but both pains are prolonged in *Gpr37*^−/−^ mice. Interestingly, zymosan-induced paw swelling (edema) is comparable in WT and *Gpr37*^−/−^ mice, peaking in 4 h but resolving after 24 h in both WT and knockout mice. Therefore, GPR37 only regulates certain aspects of inflammation and plays a specific role in resolving pain after inflammation. 

It was generally believed that macrophages produce pain by producing pro-inflammatory cytokines and chemokines (e.g., TNF, IL-1β, CCL2, CXCL2) [58,59]. Recent studies pointed to a role of macrophages in the resolution of pain [27,35,60,61]. We found that depletion of macrophages via macrophage toxin clodronate significantly reduced the number of macrophages in the inflamed skin. Furthermore, this toxin prolonged the duration of inflammatory pain, mimicking the behavioral phenotypes observed in *Gpr37*^−/−^ mice. Intriguingly, adoptive transfer of WT macrophages by intraplantar injection is sufficient to rescue the pain resolution deficits in *Gpr37*^−/−^ mice. Mechanistically, this beneficial action of macrophages is mediated by IL-10, a prominent anti-inflammatory cytokine. Thus, IL-10 administration reduced inflammatory pain, whereas the anti-IL-10 neutralizing antibody impaired the resolution of inflammatory pain [35]. 

## 4. GPR37 in Bacteria and Parasite Infections and Infection-Induced Pain

Inflammation also occurs as a result of infections, leading to an inflammatory cascade that aims to restore the homeostasis of the organism. It is crucial to precisely control this inflammatory process: either hypo- or hyper-activation of the immune system can be detrimental. A failure to mount an effective immune response to a localized infection can result in systemic inflammation, sepsis, and death [62]. Sepsis was estimated to affect nearly 50 million people worldwide in 2017. The death toll from sepsis could be as high as 30%, with over 10 million deaths [63]. It is a real medical challenge to treat sepsis, and even in high income countries, the mortality rate could reach 20%. Sepsis is driven chiefly by inflammation-induced cytokine storm and has severe consequences, such as core temperature dysregulation (fever followed by hypothermia), multiple organ failure, and septic death [64]. It has been shown that SPMs, such as resolvin D2, can effectively control bacteria-induced infections in animal models [65]. Moreover, NPD1 was shown to enhance host defense against infections of viruses, such as lethal influenza virus [66]. 

NPD1 has been shown to confer protection against bacterial infections, and furthermore, this protection is mediated by GPR37 and macrophages [36]. Intraperitoneal injection of listeria (*Listeria monocytogenes*, *L.m.*, a gram-positive bacterium) resulted in a high mortality in WT mice (~90%), associated with hypothermia and cytokine storm (surge of IL-6). Strikingly, NPD1 treatment was able to prevent listeria-induced death, hypothermia, and IL-6 surge. But this protection is lost in *Gpr37^−/−^* mice. NPD1 is also highly effective in protecting against sepsis in another commonly used model induced by lipopolysaccharide (LPS) [36]. 

GPR37 and macrophages further show protection against sepsis following malaria. It was estimated that malaria causes more than 400,000 death every year (https://www.who.int/publications-detail/world-malaria-report-2019, accessed on 14 November 2021). We identified a novel immunotherapy mechanism for malaria by which activation of GPR37 in macrophages can promote phagocytosis of parasite-infected red blood cells [36]. Intriguingly, we found artesunate (ARU), an artemisinin derivative, to be a possible ligand of GPR37. ARU is the first-line drug for the treatment of severe malaria [67]. ARU was shown to interact with GPR37 at multiple sites to form hydrogen bonds (Figure 3B). As expected, ARU treatment increased macrophage phagocytosis via GPR37, although its potency is lower than NPD1 (30 μM vs. 30 nM). Macrophages also play a protective role in fighting against malaria-induced sepsis. Depletion of macrophages is detrimental in that it increases mortality, hypothermia, and the percentage of the infected red blood cells in parasite-infected animals. In contrast, adoptive transfer of GPR37-activated macrophages by ARU or NPD1 significantly alleviated sepsis and malaria infection. Additionally, ARU substantially reduced bacterial load after infections through macrophage activation, in further support of the role of ARU in immunotherapy. Similarly, osteocalcin, a newly identified ligand of GPR37, can protect against detrimental effects caused by LPS in WT mice but not in *Gpr37^−/−^* mice. Like NPD1 and ARU, osteocalcin also triggered intracellular responses in macrophages via GPR37, altering the release of inflammatory factors and regulating macrophage phagocytosis [68]. 

Infections can cause severe pain, as some bacterial products can cause direct activation of sensory neurons, including nociceptors [6,7,69,70]. Infections further promote pain via indirect activation of the immune system [71]. Some viral infections (e.g., HIV, herpes simplex virus, and varicella zoster virus) are correlated with chronic pain and result in peripheral neuropathy [72]. Interestingly, ARU treatment rapidly reduced pain after bacterial infection, which is GPR37-mediated. Intraplantar injection of listeria induced robust mechanical and thermal pain in WT mice. Remarkably, mechanical pain resolved within two weeks in WT mice but failed to resolve in *Gpr37* knockout mice. However, adoptive transfer of ARU-stimulated peritoneal macrophages is sufficient to rescue this pain deficit. Follow-up studies showed that ARU also alleviated bone-fracture-induced pain and chemotherapy-induced pain [73,74], in further support of the pain-relieving effects of this anti-malaria drug. Thus, GPR37 agonists can be developed for the management of acute and chronic pain after infections.

## 5. Conclusions and Future Directions 

Accumulating evidence supports a protective role of GPR37 against neurodegenerative diseases following brain trauma. GPR37 deficiency results in physiological and pathological changes in oligodendrocytes, leading to increased susceptibility to demyelination. Therefore, targeting GPR37 may offer great drug discovery opportunities [75] for treating demyelinating diseases such as multiple sclerosis [45], as well as neurological diseases such as traumatic brain injury and stroke. Since both protective and detrimental roles of GPR37 have been demonstrated (Table 1), both GPR37 agonists and antagonists could be developed to tackle different diseases. 

**Figure 5:**
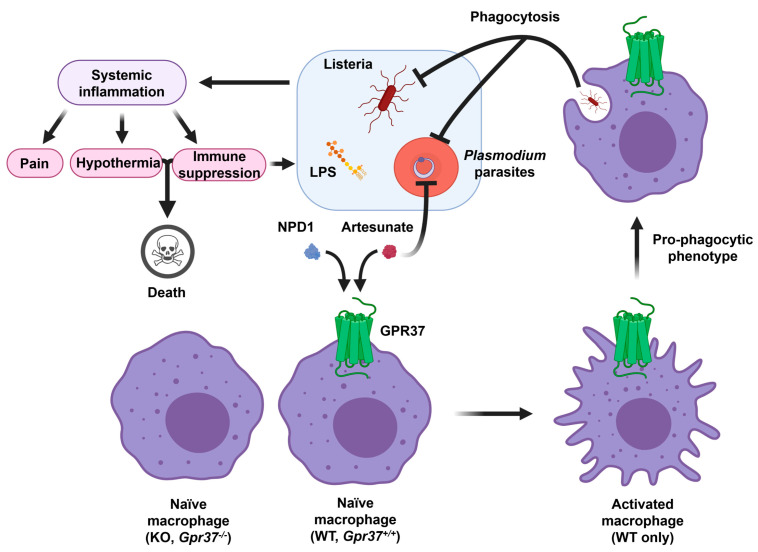
Schematic of GPR37 activation in macrophages for protection against infections. Infections by lipopolysaccharide (LPS), listeria, and parasites cause systemic inflammation, which is associated with severe pain, hypothermia, cytokine storm/immune suppression, and septic death. GPR37 activation in wild-type (WT) macrophages by NPD1 and artesunate can promote macrophage phagocytosis, leading to clearance of listeria bacteria and plasmodium-infected red blood cells. In contrast, GPR37-deficient macrophages will exacerbate inflammation, pain, and septic death. This figure is reproduced from Bang et al., Nat Commun, 2021 with permission [36]. This is an open access article under the Creative Commons CC BY license.

The resolution of inflammation is emerging as a therapeutic strategy for all the inflammation-associated diseases [15,76]. Given an important role of GPR37 in macrophage phagocytosis and inflammation resolution, GPR37 agonists could be developed to treat sepsis after bacterial and parasite infections. GPR37 ligands will also help to treat infection-induced pain that is associated with inflammation. Interestingly, both SPMs and ARU and its derivatives have been suggested as potential therapeutics for COVID through immune modulation [77]. Repurposing of antimalarial drugs (e.g., ARU) has been proposed to tackle COVID-19 [78]. As illustrated in Figure 5, infections by LPS, listeria, and parasites result in systemic inflammation, leading to pain, hypothermia, and even immune suppression. Activation of GPR37 in macrophages by NPD1 and ARU could promote macrophage phagocytosis to fight against these infections (Figure 5). 

Last but not least, chronic pain is a major health concern worldwide, affecting one-third of Americans, and pain management and labor loss cost the country $600 billion dollars per year [79]. Furthermore, lack of effective pain treatments is associated with the ongoing crisis of opioid use disorder [80]. It is striking that in 2020, more than 92,000 Americans died from drug overdoses, a nearly 30% increase over 2019, according to the Centers for Disease Control and Prevention. SARS-CoV-2 infections and long-haul COVID may further increase the incidence of chronic pain [81,82]. Thus, there is an urgent need to develop non-opioid medicine that can control excessive inflammation and neuroinflammation [82]. In particular, GPR37 agonists could be utilized to treat pain under different clinical settings, such as postoperative pain after surgery, inflammatory pain after infections and arthritis, and neuropathic pain after nerve injury, diabetes, and traumatic brain injury. In addition to macrophages, GPR37 is also expressed by primary sensory neurons. It will be of great interest to investigate whether activation of GPR37 in sensory neurons can protect against neurodegeneration after neuropathy. 

## Data Availability

Not applicable.
